# Comparison of Metabolic and Hormonal Profiles between Low-Advanced Glycation End Products (AGEs) and Standard AGEs-Containing Weight-Loss Diets in Overweight Phenotype-A PCOS Patients: A Randomized Clinical Trial

**DOI:** 10.1007/s43032-025-01808-8

**Published:** 2025-02-14

**Authors:** Merve Ozdemir, Sezcan Mumusoglu, Pelin Bilgic

**Affiliations:** 1 Department of Nutrition and Dietetics, Faculty of Health Sciences, Afyonkarahisar Health Science University, Afyon, Turkey; 2 Department of Obstetrics and Gynecology, School of Medicine, Hacettepe University, Ankara, Turkey; 3 Department of Nutrition and Dietetics, Faculty of Health Sciences, Hacettepe University, Ankara, Turkey

**Keywords:** Advanced glycation end products, Diet, Polycystic ovary syndrome, Phenotype-A, Weight loss

## Abstract

This study aims to investigate the effects of a low-advanced glycation end products(AGEs) diet versus a standard AGE-containing weight-loss diet on metabolic and hormonal profiles of overweight phenotype-A polycystic ovary syndrome(PCOS) patients.A randomized controlled interventional study.A total of 44 Rotterdam phenotype-A PCOS patients aged 19–35 were enrolled between January 2022 and May 2023. They were randomly assigned to 12-weeks of an energy-restricted Standard-AGEs diet(S-AGEs) or an energy-restricted Low-AGEs diet(L-AGEs). At baseline and after 12-weeks of intervention, weight loss, oligo-amenorrhea, hormonal profiles, plasma lipid profiles, and inflammation markers were evaluated. During the intervention, 8 participants from the L-AGEs group and 6 from the S-AGEs group dropped out. Completers had similar baseline characteristics to dropouts. In the per-protocol analysis, similar weight loss was observed in the L-AGEs(n = 14) and S-AGEs(n = 16) groups compared to baseline weight [-8.4 [-10.3 to -5.8] vs. -5.2 [-8.8 to -4.6] kg, respectively, p = 0.183]. However, in the L-AGEs group, fasting glucose levels decreased significantly more compared to the S-AGEs group (-8.5 [-11.5 to -3.5] vs. -0.5 [-3.7 to 0.7] mmol/L, respectively, p = 0.027). Following the diet intervention in the L-AGEs group, the waist-to-hip circumference ratio, LDL-cholesterol, TNF-α, total testosterone (TT), free-androgen index (FAI), and anti-Müllerian hormone (AMH) levels significantly decreased compared to baseline levels, while sex hormone-binding globulin (SHBG) levels increased. In contrast, there was no statistically significant change in these parameters in the S-AGEs group.In addition to weight-loss, reducing dietary AGEs intake resulted in significantly greater improvements in metabolic and hormonal profiles among phenotype-A PCOS patients. Clinicaltrials.gov registration no. NCT05830487.

## Introduction

Polycystic ovary syndrome (PCOS) is a heterogeneous endocrine-metabolic disorder, affecting of reproductive-aged woman, with a prevalence ranging from 10–13% [1, 2]. The main features of PCOS are hyperandrogenism, ovulatory dysfunction, and/or polycystic ovarian morphology [3, 4]. The most common symptoms of PCOS include hirsutism, oligomenorrhea, insulin resistance, obesity, and metabolic syndrome [5]. PCOS is frequently accompanied by overweight and obesity, which exacerbates symptoms related to the reproductive system, metabolism, such as decreased ovulation frequency, irregular menstrual cycles, impaired fertility, and hyperandrogenemia [6]. Losing weight is advised as a first-line therapies since it can improve metabolic symptoms like insulin resistance and risk factors for cardiovascular disease and type 2 diabetes, as well as reproductive function [1]. Lifestyle changes, aiming for a 5% to 10% weight loss, a low-glycemic index and hypocaloric diet, and regular exercise are first-line therapies [2, 7]. Traditionally, diet interventions for PCOS have focused on nutrients and calories. However, research in the last times have suggested that chronic inflammation and increased oxidative stress may contribute to the pathophysiology of PCOS through AGEs mechanisms of action [5, 8–10]. Chronic inflammation and elevated oxidative stress have been proposed as potential links between AGEs action mechanisms and the metabolic and reproductive implications of PCOS.

A diet rich in AGEs may contribute to reproductive and metabolic abnormalities in PCOS [11]. Studies show higher serum AGEs levels in women with PCOS [9, 10]. AGEs are compounds formed by the non-enzymatic interaction of reducing sugars and amino groups. They act via receptor-dependent pathways, attaching to RAGE receptors on cell membranes, and receptor-independent pathways. RAGE is expressed in the reproductive system and other tissues. Elevated AGEs trigger MAP kinase, NF-κB, and other critical cellular signaling routes, leading to cellular malfunction and various diseases [5, 12]. AGEs originate from endogenous formation during metabolism and from diet [13]. About 10% of dietary AGEs are absorbed, with two-thirds stored in tissues and one-third eliminated [14]. High tissue and blood AGEs levels are pathogenic, increasing oxidative stress and inflammation by binding to cell surface receptors or cross-linking with body proteins, altering their structure and function [15, 16]. AGEs are abundant in animal-derived foods and increase significantly with high-temperature cooking methods like grilling and roasting. The Western diet, high in animal products and fast foods, is a major AGEs source [15, 17].

Rats fed a high-AGEs diet for six months accumulated AGEs in the reproductive system and had increased plasma testosterone levels [18]. Similarly, women with PCOS on a high-AGEs diet for two months showed higher testosterone levels and free androgen index compared to those on a low-AGEs diet [19].

This randomized control study aimed to compare the effects of an energy-restricted diet and an energy-restricted diet with reduced AGEs on weight loss, oligo-amenorrhea, hormonal profile, plasma lipid profile, and inflammation markers in women with PCOS.

## Materials and Methods

### Study Population

The study was conducted at Hacettepe University between January 2022 and May 2023 in collaboration with the Department of Obstetrics and Gynecology and the Department of Nutrition and Dietetics. The study's inclusion criteria were as follows: i) participants aged between 19 and 35 years, ii) diagnosed with phenotype-A PCOS according to Rotterdam criteria (patients who have all three features of PCOS: hyperandrogenism, oligo anovulation, and polycystic ovarian morphology) [20], and iii) body mass index (BMI) > 25 kg/m^2^. The exclusion criteria were a history of any metabolic syndrome, active smoking, use of medications known to affect glucose metabolism or lipid profile, use of vitamin supplements, major food allergies, history of eating disorders, significant weight loss, or changes in dietary habits within the previous three months, and use of oral contraceptives. Additionally, the study did not include pregnant or breastfeeding women.

Before enrollment, all patients were given details about the study's objectives, protocol, and methodology. After being informed, they voluntarily signed a written consent form. The Ministry of Health and the Clinical Research Ethics Boards of Hacettepe University (research ethics project number, KA-21096) approved the study.

### Study Design

**Fig. 1:**
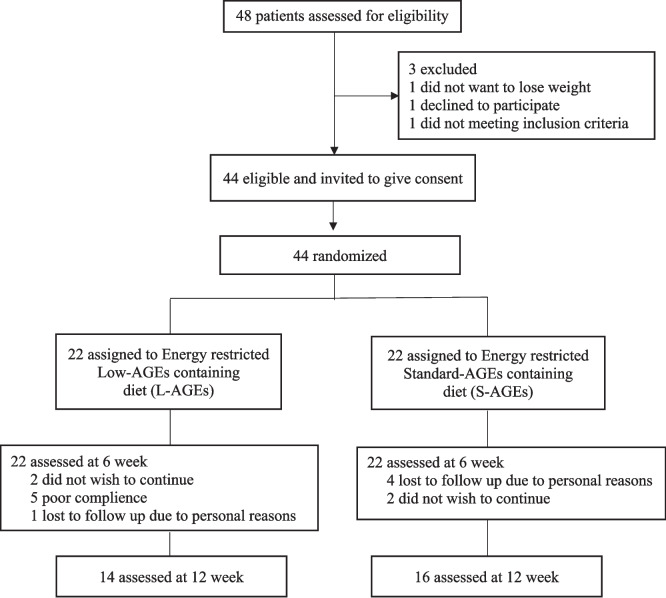
Trial flow diagram

The study was a two-arm parallel, randomized controlled trial. Each participant followed one of two dietary treatments for 12 weeks. Participants assigned via stratified randomization based on age and BMI. Before the study began detailed case reports were collected through face-to-face interviews, covering general information, nutritional habits, physical activity, anthropometrics, health status, and a 3-day food intake records. Participants were instructed to maintain their physical activity levels and adhere to the dietary guidance provided. They received training specific to their dietary plans. Compliance with the interventions and measurements was assessed through interviews in the 6th and 12th weeks. The trial's flow diagram is shown in Fig. 1.

### Dietary Intervention

In this study, intervention diets were divided into two categories: “energy restricted Low-AGEs containing diet” and “energy restricted Standard-AGEs containing diet.” Energy requirements participants were computed using the Harris-Benedict formula. The energy content of the diets was customized based on calculated energy requirements and participants' physical activity levels.

Energy restricted Low-AGEs containing diet (L-AGEs): Alongside the standard energy restricted diet typically administered, this dietary approach emphasizes cooking methods such as boiling and stewing instead of deep-frying and roasting. Patients in this category were provided with guidance on food preparation, including instructions on suitable cooking durations and temperatures. They were given instructions such as boil, steam, and marinade foods (with lemon or vinegar before cooking). Instructions included avoiding industrially processed items known to be baked at high temperatures, such as cereals and cookies. Regular interviews with the researcher’s were undertaken twice a week to ensure that patients followed the diet.

Energy restricted Standard-AGEs containing diet (S-AGEs): Weight loss is recommended as first-line treatment for overweight PCOS patients. An important guideline [3] for is to aim for a weight reduction of at least 7%, targeting obesity as a risk factor for PCOS. This dietary approach for weight loss (5–10%) by reducing a patient's required energy intake (500–1000 kcal/day), is structured so that fat accounts for 20–25% of total energy consumption. All patients in this group were given instructions to continue their usual cooking methods.

### Antropometric Mesurements

At the onset and conclusion of the intervention, researcher’s dietitian performed all anthropometric assessments on the participants at the Anthropometry Laboratory within the Department of Nutrition and Dietetics at Hacettepe University. Body weight and composition were evaluated using a Tanita MC980 MA (Tanita, Tokyo, Japan). Height was measured using a stadiometer, with individuals standing barefoot and aligning their head with the Frankfurt plane. Body mass index (BMI) was computed by dividing weight (kg) by the square of height (m^2^). Waist circumference was evaluated by placing a tape measure at the midpoint between the lowest rib bone and the crista iliac bone. Hip circumference was measured at the widest part across the buttocks, and then the waist-to-hip ratio was computed. Visceral and abdominal adiposity were assessed using the abdominal BIA equipment ViScan (Tanita AB-140, Tokyo, Japan), which is specifically designed to determine visceral adiposity and trunk fat percentage while the patient is supine.

### Assessment of Dietary AGEs Intake

Before and after the trial 3-day (2 weekdays and 1 weekend day) detailed dietary records were obtained from participants. These records contained details regarding cooking methods and portion sizes for consumed food items. The nutritional content of foods was assessed utilizing The Nutrition Information System (BeBis) 8.2 full version software. Data regarding the AGEs content in foods were retrieved from the Carboxymethyl Lysine (CML) database, as published by Uribarri et al. [15]. This database contains data on 549 items, including the most popular foods and widely utilized cooking methods. It is crucial to note that this database may not contain all of the food items from the consumption records. The AGEs content of foods not included in the database were assessed in comparison to similar food items, considering their nutritional and compositional features.

### Blood Sample Collection and Analysis

Blood samples were drawn into serum clot activator blood tubes from volunteers who applied to the clinic and met the study criteria after a 12-h fasting. Before being centrifuged, serum tubes were left to coagulate at room temperature for sixty minutes. The tubes were centrifuged for 10 min at 4 °C at 1000 × g. After that, the serum was put into a sterile polypropylene tube, frozen at −80 °C, and kept for batch analysis. Circulating AGEs (CML, sRAGE) and inflammation indicators (TNF-a, hs-CRP) levels were measured using the enzyme-linked immunosorbent assay (ELISA) method in serum samples collected at the beginning and end of the study. The Bioassay Technology Laboratory (BT-Lab; Shanghai, China) Human CML ELISA kit (Cat. No. E1413Hu, sensitivity: 9.52 ng/mL, detection range from 20–3000 ng/mL), Human sRAGE ELISA kit (Cat. No. E0027Hu, sensitivity: 0.01 ng/mL, detection range from 0.05–20 ng/mL), Human TNF-ELISA kit (Cat. No. E0082Hu, sensitivity: 1.52 ng/mL, detection range from 3–900 ng/mL), Human hs-CRP ELISA kit (Cat. No. E1805Hu, sensitivity: 0.053 ng/mL, detection range from 0.1–40 ng/mL). Total antioxidant status (TAS) levels were tested with commercial kits (Relassay, Turkey). The assay provides good precision values that are less than 3%. The results were represented in mmol Trolox equivalent per liter. Total oxidant status (TOS) levels were tested with commercially accessible kits (Relassay, Turkey). The assay was calibrated using hydrogen peroxide, and the results were represented in micromolar hydrogen peroxide equivalent per liter (μmol H_2_O_2_/L). The ratio of TOS to TAS was accepted as the oxidative stress index (OSI) [21]. For calculation, the resulting unit of TAS was converted to μmol/L, and the OSI value was calculated according to the following Formula: OSI (arbitrary unit) = TOS (μmol H2O2 equivalent/L) / TAC (μmol Trolox equivalent/L).

Fasting insulin, fasting glucose, lipid parameters (triglyceride, LDL-C, HDL-C, total cholesterol), total testosterone (TT), sex hormone-binding globulin (SHBG), dehydroepiandrosterone sulfate (DHEA-SO4) and Anti-mullerian hormone (AMH) values were recorded from the patients' files before to and following the dietary intervention. TT and DHEA-SO4 levels were measured by the chemiluminescence microparticle enzyme immunoassay method (Siemens Healthineers, Advia Centaur CP Immunoassay System, Germany), and SHBG, AMH and insulin levels were measured by chemiluminescent microparticle enzyme immunoassay method (Beckman Coulter, UniCel DxI 800 Access Immunoassay System, USA). Lipid profile and glucose levels were measured by a single-step immunoenzymatic method (Beckman Coulter, Inc., USA). Free androgen index (FAI) and homeostasis model assessment index-insulin resistance (HOMA-IR) measurements were calculated according to standard accepted equations. (FAI = [Total testosterone (mmol/L) / SHBG (nmol/L)*100]), HOMA-IR = [fasting insulin ((U/mL)*fasting plasma glucose (FPG) (mg/dL) / 405]).

### Statistical Analysis

The statistical analysis was carried out using IBM SPSS Statistics Software for Windows version 26.0 (IBM Corporation, Armonk, New York, NY, USA). The sample size was calculated using G*Power analysis software based on the predicted change in serum CML levels following the AGE-restricted diet [22], resulting in a total of 24 participants (12 in each group). Before analysis, the Kolmogorov–Smirnov test was used to determine the normality of the data, which revealed that the data were not normally distributed. As a result, all data were reported as the median and quartiles. The Wilcoxon rank test was used to compare plasma values before and after intervention, and the Mann–Whitney U test was used to compare the two diets. "We used the CONSORT checklist when writing our report [23]".

## Results

A total of 47 women with phenotype A PCOS were initially assessed; however, three patients were excluded for not meeting the inclusion criteria. Of the remaining participants, 22 women were assigned to the S-AGEs diet group and 22 to the L-AGEs diet group. The baseline demographic and menstrual cycle characteristics of both groups are presented in Table 1. There were no statistical differences in factors such as age [26.5 (23.0–29.5) vs. 25.5 (23.5–27.2), p = 0.364], BMI [37.7 (33.2–39.7) vs. 37.4 (32.6–40.3), p = 0.935) and hirsutism score [15.5 (7.7–24.0) vs. 16.0 (11.2–22.7), p = 0.796] between participants in both diet groups. After the 6-weeks intervention period, 8 participants from the L-AGEs group, ( 2 did not wish to continue, 5 poor complience, 1 personal reasons) and 6 participants from the S-AGEs group (4 personal reasons, 2 did not wish to continue) dropped out of the study. Consequently, the L-AGEs group was completed with 14 participants, whereas the S-AGEs group was completed with 16 participants (Fig. 1). Participants who completed the intervention had the same baseline age, body weight, BMI, and hirsutism scores as those who dropped out, as shown in Table 2.

**Table 1:** Comparisons of baseline demographic and menstrual cycle characteristics among the initially recruited study population in each diet group

Characteristics	L-AGEs (n = 22)	S-AGEs (n = 22)	*p*
Age (y)	26.5 (23.0–29.5)	25.5 (23.5–27.2)	0.364
Body weight (kg)	99.4 (83.2–109.0)	94.7 (81.7–108.5)	0.432
BMI (kg/m^2^)	37.7 (33.2–39.7)	37.4 (32.6–40.3)	0.935
Education Level* n* (%)			
≤ High school	12 (55)	6 (27)	
Bachelor	9 (41)	14 (64)	
Master/Doctorate	1 (4)	2 (9)	
Physical activity (METs-min/week)	912.0 (691.0–1155.0)	855.0 (693.0–990.0)	0.459
Hirsutism score (*m-*FG-Score)	15.5 (7.7–24.0)	16.0 (11.2–22.7)	0.796
Age at menarche (y)* n (%)*			
≤ 11	1 (4)	4 (18)	
12–15	21 (96)	18 (82)	
Duration of menstrual bleeding (day)	6.0 (5.0–7.0)	5.0 (5.0–7.0)	0.448
Median duration of menstrual cycle (day)	60.0 (40.0–75.0)	60.0 (40.0–90.0)	0.666

Values are presented as median (25th-75th percentile) or *n* (%). L-AGEs = Energy restricted Low-AGEs containing diet; S-AGEs = Energy restricted Standard-AGEs containing diet; BMI = Body mass index; METs = Metabolic equivalent. mFG = modified Ferriman Gallawey score, *p:* difference in mean change between L-AGEs vs. S-AGEs groups. *Mann Whitney U test*

**Table 2:** Comparisons of baseline demographic and menstrual cycle characteristics between patients who completed the study and those who dropped out in each diet group

Characteristics	L-AGEs (n = 14)	Drop-out in L-AGEs (n = 8)	*p*	S-AGEs (n = 16)	Drop-out in S-AGEs (n = 6)	*p*
Age (y)	25.0 (23.0–28.0)	28.00 (24.0–32.0)	0.193	25.0 (24.0–27.0)	27.0 (21.5–29.7)	0.373
Body weight (kg)	98.0 (82.7–112.8)	101.7 (86.0–107.2)	0.733	89.4 (80.1–101.9)	107.7 (100.6–110.6)	0.106
BMI (kg/m^2^)	37.5 (33.2–40.2)	37.7(33.0–38.3)	0.733	36.1(32.0–40.2)	39.3(36.2–40.6)	0.210
Education Level *n* (%)						
≤ High school	7 (50)	4 (50)		3 (19)	3 (50)	
Bachelor	6 (43)	4 (50)		11 (69)	3 (50)	
Master/Doctorate	1 (7)	-		2 (12)	-	
Physical activity (MET-min/wk)	912.0 (737.5–1031.3)	907.5 (618.7–1196.3)	0.891	912.0 (693.0–1012.0)	752.5 (576.5–949.5)	0.319
Hirsutism score (*m-*FG-Score)	15.5(7.0–23.0)	19.5(11.0–28.2)	0.373	15.5 (9.0–24.2)	17.5 (14.7–22.7)	0.437
Age at menarche (y) *n* (%)						
≤ 11	1 (7)	-		2 (13)	2 (33)	
12–15	13 (93)	8 (100)		14 (87)	4 (67)	
Duration of Menstrual bleeding (day)	6.0 (5.0–7.0)	5.5 (5.0–6.7)	0.354	6.5 (5.0–7.0)	5.0 (5.0–5.2)	0.219
Median duration of menstrual cycle (day)	60.0 (43.7–86.2)	60.0 (36.0–71.2)	0.357	60.0 (40.0–90.0)	40.5 (33.7–82.5)	0.231

Values are presented as median (25th-75th percentile) or *n* (%). L-AGEs = Energy restricted Low-AGEs containing diet; S-AGEs = Energy restricted Standard-AGEs containing diet; BMI = Body mass index; METs = Metabolic equivalent. *m-*FG = modified Ferriman Gallawey score, *p* = difference in mean change, *Mann Whitney U test*

The body composition, energy levels, and AGEs values of the groups at the beginning and after the 12-week follow-up are shown in Table 3. Weight loss [−8.4 [−10.3-(−5.8)] vs. −5.2 [−8.8-(−4.6)] kg, p = 0.001], BMI [−3.2 [−4.0-(−2.0)] vs. −2.2 [−3.4-(−1.8)], p = 0.001], visceral fat [−2.4 [−4.4- (1.8)] vs. −2.0 [−3.6-(−1.1)], p = 0.001], and body fat percentage [−2.2 [−3.3-(−1.3)] vs. −2.2 [−3.3-(−0.9)], p = 0.001] were all significantly reduced in both diet groups. There was no statistically significant difference between diet groups in terms of reductions weight loss (p = 0.183), BMI (p = 0.739), visceral fat (p = 0.188) and body fat percentage (p = 0.662). However, L-AGEs group exhibited a significantly lower waist-hip ratio [0.85 (0.80–0.92) vs. 0.84 (0.78–0.90), p = 0.009]. In contrast, there was no statistically significant change in this parameter in the S-AGEs group. Modified Ferriman Gallawey (m-FG) scores was slightly, but not substantially, reduced in the L-AGEs group [15.5 (7.0–23.0) vs. 14.0 (7.0–23.0), p = 0.063], but not S-AGEs group [15.5 (9.0–24.2) vs. 15.5 (9.0–23.5), p = 0.317]. The number of regular menstrual cycles was increased in 71% (10/14) of patients in the L-AGEs group and 50% (8/16) of patients in the S-AGEs group in comparison with baseline. But there was no statistical difference between the two groups (p = 0.206).

**Table 3:** Comparison of body composition, energy and AGEs values between groups at baseline and 12-wk follow-up

	L-AGEs (n = 14)				S-AGEs (n = 16)				
Variables	Baseline	12 wk	Δ	p^*^	Baseline	12 wk	Δ	p^*^	p**
Weight (kg)	98.0 (82.7–112.8)	87.8 (79.7–104.6)	−8.4 [−10.3-(−5.8)]	0.001	89.4 (80.1–103.3)	85.5 (75.0–89.1)	−5.2 [−8.8-(−4.6)]	0.001	0.183
BMI (kg/m^2^)	37.5 (33.2–40.2)	34.4 (31.4–36.4)	−3.2 [−4.0-(−2.0)]	0.001	36.1 (32.0–40.2)	33.9 (30.2–35.3)	−2.2 [−3.4-(−1.8)]	0.001	0.339
Waist-to-hip ratio	0.85 (0.80–0.92)	0.84 (0.78–0.90)	−0.01 (−0.027–0.007)	0.009	0.83 (0.81–0.90)	0.82 (0.79–0.88)	−0.004 (−0,13–0.005)	0.088	0.739
Body fat, %	41.7 (37.1–43.9)	37.7 (35.1–42.1)	−2.2 [−3.3-(−1.3)]	0.001	37.1 (35.7–43.1)	37.8 (33.9–39.7)	−2.2 [−3.3-(−0.9)]	0.024	0.662
Trunk %Fat	48.8 (44.5–53.4)	47.0 (41.8–50.0)	−2.0 [−2.4-(−1.1)]	0.001	44.0 (41.8–50.8)	42.9 (39.3–46.8)	−1.2 [−1.8-(−0.7)]	0.001	0.313
Visceral fat level	13.5 (11.8–18.5)	12.5 (9.8–16.3)	−2.4 [−4.4- (1.8)]	0.001	11.3 (9.5–15.9)	10.5 (8.6–14.1)	−2.0 [−3.6-(−1.1)]	0.001	0.188
Hirsutism score (*m-*FG-Score)	15.5 (7.0–23.0)	14.0 (7.0–23.0)	0.0 (0.0–1.0)	0.063	15.5 (9.0–24.2)	15.5 (9.0–23.5)	0.0 (0.0–0.0)	0.317	0.096
Dietary energy (kkal/day)	1957.7 (1759.6–2573.5)	1511.8 (1471.4–1537.7)	−418.4 [−1036.7-(−261.0)]	0.001	2223.3 (1792.4–2593.7)	1552.0 (1509.3–1605.9)	−636.1 [−1085.4-(−171.8)]	0.000	0.803
Dietary AGEs (kU/day)	15,981.5 (14,484.5–18,137.5)	6375.0 (5648.2–6910.5)	−9961.0 [−11523.3-(−7930.8)]	0.001	16,514.5 (14,462.0–20365.7)	16,410.0 (14,211.7–17,788.7)	−1536.5 (−3889.0–1920.7]	0.278	0.000
Serum CML (ng/mL)	687.1 (405.3–2766.5)	543.6 (340.2–2322.0)	−128.8 [−426.5-(−62.0)]	0.03	437.8 (275.5–1620)	546.8 (333.4–1728.2)	125.1 (−81.6–247.8)	0.03	0.002
Serum sRAGE (ng/mL)	3.1 (1.7–19.6)	4.6 (2.4–23.3)	0.93 (0.58–3.7)	0.006	5.7 (3.4–18.5)	4.9 (2.0–16.5)	−1.5 (−2.7- (−0.1)	0.005	0.000

All values expressed as median (25th-75th percentile). L-AGEs = Energy restricted Low-AGEs containing diet; S-AGEs = Energy restricted Standard-AGEs containing diet; BMI = Body mass index;

AGEs = Advanced glycation end products; CML = N-carboxymethyl lysine; sRAGE = Soluble receptor for advanced glycation end products. p^*^ = change between baseline and 12 week within group, Wilcoxon Test. p^**^ = difference in mean change between L-AGEs vs. S-AGEs groups, *Mann whitney U test*

During and after the study, patients in the L-AGEs group consumed over 60% less dietary AGEs than those in the S-AGEs group. Serum CML levels decreased dramatically in the L-AGEs group [687.1 (405.3–2766.5) vs. 543.6 (340.2–2322.0) ng/mL, p = 0.03] but increased significantly in the S-AGEs group [437.8 (275.5–1620) vs. 546.8 (333.4–1728.2) ng/mL, p = 0.03]. The difference between the two diet groups is statistically significant (p = 0.002). Serum sRAGE levels increased statistically significantly in the L-AGEs group [3.1 (1.7–19.6) vs. 4.6 (2.4–23.3) ng/mL, p = 0.006] but decreased statistically significantly in the S-AGEs group [5.7 (3.4–18.5) vs. 4.9 (2.0–16.5) ng/mL, p = 0.005] with a statistical difference between diet groups (p < 0.001).

Clinical and biochemical characteristics of the groups at baseline and 12-weeks of follow-up were shown in Table 4. In both groups, fasting insulin and HOMA-IR levels decreased significantly (p < 0.005). There was a statistically non-significant decrease in hs-CRP levels [6.8 (4.4–9.2) vs. 5.4 (4.1–9.5) ng/mL, p = 0.124] but a statistically significant decrease in TNF-a values [128.5 (88.4–182.4) vs. 116.9 (63.5–157.2) ng/L, p = 0.004] and LDL cholesterol [133.5 (127.0–160.5) vs. 131.0 (119.7–149.7) mg/dL, p = 0.046) in the L-AGEs group. But, there was no statistically significant changes in these parameters in the S-AGEs group. Although there was no statistically significant changes in TAS and TOS levels in the L-AGEs group, a significant decline in TAS level [1.8 (1.6–2.1) vs. 1.7 (1.5–1.8) mmol/L, p = 0.013] was noted in the S-AGEs group.

**Table 4:** Comparison of clinical and biochemical characteristics between groups at baseline and 12-wk follow-up

	L-AGEs (n = 14)				S-AGEs (n = 16)				
Variables	Baseline	12 wk	Δ	p^*^	Baseline	12 wk	Δ	p^*^	p^**^
Fasting glucose (mmol/L)	89.0 (84.0–95.5)	81.5 (77.2–90.5)	−8.5 [−11.5-(−3.5)]	0.064	87.0 (78.2–92.0)	86.5 (78.0–92.7)	−0.5 (−3.7–0.7)	0.346	0.027
Fasting insulin (pmol/L)	11.7 (8.4–13.6)	8.2 (6.3–11.2)	−2.7 [−5.2-(−1.6)]	0.002	11.6 (7.0–24.6)	10.7 (5.3–16.3)	−2.3 (−8.4–0.27)	0.027	0.678
HOMA-IR	2.38 (1.86–2.99)	1.84 (1.32–2.59)	−0.8 (−1.4–0.1)	0.016	2.74 (1.50–5.61)	2.34 (1.20–3.62)	−0.5 [−2.3-(−0.3)]	0.023	0.948
QUİCKİ	0.33 (0.32–0.34)	0.35 (0.32–0.36)	0.02 (−0.02–0.04)	0.041	0.33 (0.30–0.36)	0.34 (0.32–0.37)	0.01 (0.00–0.03)	0.053	0.567
Total cholesterol (mg/dL)	198.0 (171.7–228.2)	194.0 (179.0–216.2)	−4.0 (−20.0–7.0)	0.451	171.5 (160.7–200.5)	170.0 (146.2–207.0)	−3.0 (−18.2–22.3)	0.877	0.771
LDL-C (mg/dL)	133.5 (127.0–160.5)	131.0 (119.7–149.7)	−1.5 (−12.7–0.5)	0.046	115.5 (99.7–126.7)	108.5 (92.7–144.2)	−1.5 (−14.5–18.2)	0.856	0.479
HDL-C (mg/dL)	43.5 (39.5–56.2)	45.5 (39.7–56.7)	−1.6 (−3.2–8.0)	0.753	47.0 (38.2–54.0)	46.5 (45.0–53.2)	−0.5 (−3.5–2.7)	0.909	0.884
VLDL-C (mmol/L)	20.5 (15.5–31.2)	23.5 (15.7–29.5)	−0.5 (−2.0–4.7)	0.599	23.5 (14.6–37.0)	20.0 (12.0–34.7)	−2.0 (−7.4–0.7)	0.118	0.113
Triglycerides (mg/dL)	104.0 (78.0–155.2)	118.5 (78.5–149.0)	−5.5 (−10.5–24.2)	0.683	116.0 (71.0–184.0)	104.5 (61.0–174.2)	−7.5 (−35.0–7.5)	0.173	0.197
hs-CRP (ng/mL)	6.8 (4.4–9.2)	5.4 (4.1–9.5)	−0.06 (−1.4–0.2)	0.124	9.8 (6.6–23.6)	10.0 (6.2–23.5)	0.9 (−0.5–2.3)	0.301	0.053
TNF-α (ng/L)	128.5 (88.4–182.4)	116.9 (63.5–157.2)	−24.7 [−42.0-(−13.1)]	0.004	136.0 (115.9–210.0)	133.4 (72.9–188.3)	−20.1 (−70.5–12.8)	0.063	0.934
TAS (mmol/L)	1.8 (1.6–2.0)	1.7 (1.6–1.9)	−0.1 (−0.3–0.1)	0.109	1.8 (1.6–2.1)	1.7 (1.5–1.8)	−0.1 (−0.4–0.02)	0.013	0.603
TOS (µmol/L)	4.2 (3.3–5.6)	4.0 (3.4–5.9)	−0.3 (−1.5–1.2)	0.683	5.1 (4.2–5.6)	4.0 (3.4–5.7)	−0.2 (−2.5–0.9)	0.408	0.708
OSI (arbitrary unit)	0.23 (0.19–0.3)	0.22 (0.2–0.35)	−0.0 (−0.08–0.05)	0.683	0.26 (0.22–0.32)	0.24 (0.17–0.39)	−0.0 (−0.09–0.08)	0.756	0.739

All values expressed as median (25th-75th percentile).L-AGEs = Energy restricted Low-AGEs containing diet; S-AGEs = Energy restricted Standard-AGEs containing diet; HOMA-IR = Homeostatic model assessment of insulin resistance; QUİCKİ = Quantitative insulin sensitivity check index; LDL-C = Low-density lipoprotein cholesterol; HDL-C = High-density lipoprotein cholesterol; VLDL-C = Very low-density lipoprotein cholesterol; Hs-CRP = *high*-*sensitivity* C-reactive protein; TNF-a = Tumor necrosis factor-alpha; TAS = Total antioxidant status; TOS = Total oxidant status; OSI = Oxidative stress index p^*^ = change between baseline and 12 week within group,Wilcoxon Test; p^**^ = difference in mean change between L-AGEs vs. S-AGEs groups, *Mann whitney U test*

Hormonal characteristics of the groups at the start of the study and after the 12-week follow-up are shown in Table 5. L-AGEs group showed a statistically significant increase in SHBG levels [22.5 (12.7–28.0) vs. 22.2 (15.7–31.5) nmol/L, p = 0.042] and a statistically significant decreased in total testosterone [52.2 (44.5–69.6) vs. 45.5 (27.2–52.8) ng/dL, p = 0.009] and FAI [9.03 (4.1–11.0) vs. 7.1 (3.9–8.5), p = 0.028]. There was no statistically significant changes in these parameters in the S-AGEs group. AMH levels decreased significantly in the L-AGEs group [−0.5 (−1.6–0.2) ng/mL, p = 0.041] but not in the S-AGEs group [−0.1 (−0.8–0.3) ng/mL, p = 0.514].

**Table 5:** Comparison of hormonal characteristics between groups at baseline and 12-wk follow-up

	L-AGEs (n = 14)				S-AGEs (n = 16)				
Variables	Baseline	12 wk	Δ	*p*^*a*^	Baseline	12 wk	Δ	*p*^*a*^	*p*^*b*^
Total testosterone (ng/dL)	52.2 (44.5–69.6)	45.5 (27.2–52.8)	−13.0 [−20.1-(−0.6)]	0.009	56.8 (39.9–69.1)	44.6 (32.1–62.2)	−4.6 (−24.4–7.2)	0.215	0.405
SHBG (nmol/L)	22.5 (12.7–28.0)	22.2 (15.7–31.5)	0.8 (0.0–3.5)	0.042	26.6 (18.2–33.5)	26.6 (17.8–40.0)	0.0 (−2.0–0.0)	0.285	0.266
DHEA-SO4 (µg/dL)	235.0 (160.5–316.2)	220.0 (168.8–304.7)	−8.0 (−62.2–15.9)	0.182	286.7 (198.0–359.1)	281.1 (208.6–324.4)	0.0 (−66.4–39.1)	0.272	0.726
FAI	9.03 (4.1–11.0)	7.1 (3.9–8.5)	1.2 (−3.7–0.06)	0.028	5.86 (2.85–10.17)	6.0 (2.7–11.0)	−0.1 (−1.2–2.3)	0.929	0.105
AMH (ng/mL)	6.4 (4.4–11.1)	5.9 (3.9–11.6)	−0.5 (−1.6–0.2)	0.041	6.6 (4.4–10.0)	6.8 (4.4–9.6)	−0.1 (−0.8–0.3)	0.514	0.205

All values expressed as median (25th-75th percentile) L-AGEs = Energy restricted Low-AGEs containing diet; S-AGEs = Energy restricted Standard-AGEs containing diet; SHBG = *Sex hormone-binding globulin;* DHEA-SO4 = Dehydroepiandrostenodione sulfate; FAI = Free Androgen *Index;* AMH = Anti-mullerian hormon. p^*^ = change between baseline and 12 week within group, Wilcoxon Test. p^**^ = difference in mean change between L-AGEs vs. S-AGEs groups, *Mann whitney U test*

## Discussion

While weight loss serves as the foundation for treating PCOS, we found that reducing dietary AGEs, in addition to weight loss, leads to a more significant improvement in hormonal parameters in obese women diagnosed with phenotype-A PCOS. These findings highlight the potential benefits of such dietary changes in the management of PCOS.

Obesity is a notable risk factor for both reproductive and metabolic diseases in people with PCOS. Individuals with high body fat tend to consume more calories and AGEs through their diet, which is commonly driven by the adoption of the Western-type diet that is rich in saturated fats and simple sugars [24]. This increased energy and AGEs intake, which is commonly associated with obesity and the Western-type diet, may contribute to increased oxidative stress and endogenous AGEs production. Weight management therapies based on energy-restricted diets could be an effective technique for reducing serum AGEs concentrations. These interventions have a twofold effect: they not only decrease dietary intake but also reduce the formation of endogenous AGEs, thus tackling oxidative stress in individuals [25]. A study by Diamanti-Kandarakis et al. [9], found that overweight women with PCOS had higher serum levels of AGEs than those without PCOS, regardless of the level of hyperglycemia, which is well-known to be related to an increase in AGEs levels. By the same study group, it was shown that lean women with PCOS, who did not have insulin resistance features like hyperandrogenemia [10]. Research on women undergoing in vitro fertilization (IVF) treatment reported notably reduced levels of sRAGE in follicular fluid in woman with PCOS compared to those without PCOS [26, 27]. These data indicate that there are even modifications in anti-inflammatory sRAGE receptors in women with PCOS [28]. Although certain studies [29–31] propose sRAGE concentrations as a potential biomarker in obese patients, findings are inconsistent [32, 33]. In addition to the typical receptor, variants of RAGE missing both the cytosolic and transmembrane domains have been identified. These types of AGEs receptors are produced extracellularly, detectable in circulating blood, and referred to as soluble RAGE (sRAGE). sRAGE can attach to AGEs in the bloodstream, preventing the AGE-RAGE axis's negative intracellular effects [5, 12]. In contrast to RAGE, sRAGE demonstrates an "anti-inflammatory" function by binding to circulating AGEs, thereby hindering their pro-inflammatory impact by impeding their binding to RAGE [28]. The mechanisms underlying the reduction of serum AGEs concentration and the sRAGE response to weight loss intervention remain unclear [25]. The current investigation found that serum CML were reduced while serum sRAGE increased in the L-AGEs group. Despite similar weight loss in the S-AGEs group, serum CML levels increased, but sRAGE levels decreased significantly.

Anti-mullerian hormone is a good ovarian reserve marker and plays a role during folliculogenesis. It decreases granulosa cell differentiation and inhibits follicle maturation and atresia, especially in the early phases of development [34]. Apart from increased AGEs levels, women diagnosed with PCOS frequently exhibit elevated levels of AMH, which are thought to contribute to abnormal folliculogenesis and anovulation [35]. The correlations observed among PCOS, AGEs, and AMH imply that AGEs might be associated with higher AMH production, consequently may lead to anovulation [5]. Research has indicated a relationship between follicular sRAGE and AMH levels in women of reproductive age, indicating a potential link between sRAGE and fertility [26]. The accumulation of AGEs in human ovaries may explain a number of age-related characteristics of ovarian dysfunction, including poor vascularization caused by oxidative stress and consequent hypoxia, as well as reduced nutrition uptake by granulosa cells [36]. Long-term AGEs exposure during reproductive life may result in mild oxidative damage to ovarian follicles [37]. These changes in the ovarian microenvironment may jeopardize follicular health and maturation by impairing granulosa cell metabolism, antioxidant defense production, and the establishment of sufficient perifollicular vascularization [26]. At the same time, certain studies have connected the adverse impacts of the AGE-RAGE axis on folliculogenesis and the follicular microenvironment with ovarian dysfunction and unfavorable outcomes in IVF [28, 38]. In the current study despite significant weight loss in both groups, there was no significant change in AMH levels in the S-AGEs group. However, AMH levels significantly decreased and the number of regular menstrual cycles significantly increased in the L-AGEs group. This emphasizes the importance of interventions targeting the reduction of dietary AGEs intake alongside the treatment objective of weight loss during this phase of PCOS.

Various steroidogenic enzymes in PCOS change steroid biosynthesis, resulting in increased androgen production. Hyperandrogenism is thought to contribute to PCOS-related infertility. AGEs are thought to modulate steroid production by affecting enzyme activity in polycystic ovaries [39], it has been reported that, in woman with PCOS, serum total testosterone, FAI, and androstenedione levels were considerably higher in the group ingesting an isocaloric diet with high AGEs for two months compared to women getting an isocaloric diet with low AGEs [19]. In another study conducted on rats, it was stated that AGEs increased in the theca cells of the ovarian tissue of rats fed a diet high in AGEs for six months. Furthermore, plasma testosterone levels were higher in this group compared to rats fed a low AGEs diet [18]. Rats on a high AGEs diet showed higher levels of glucose, insulin, and testosterone compared to those fed a low AGEs diet [40]. Consistent with these findings, we found that the women with Phenotype-A PCOS L-AGEs group a statistically significant decrease in serum TT and FAI levels and a statistically significant increase in SHBG levels. There was no statistically significant changed in this levels in the S-AGEs group. Disruption in both metabolic and hormonal functions may linked to the consumption of a dietary AGEs contents [40]. These findings also highlight the link between dietary AGEs and hyperandrogenemia, lending support to the idea that lowering dietary AGEs in PCOS may ameliorate some hyperandrogenemia symptoms. In this study, m-FG scores decreased slightly, although not statistically, in the L-AGEs group (p = 0.063), but not in the S-AGEs group (p = 0.317). Adopting a low-AGEs diet may serve as a feasible treatment objective to reduce risks associated with PCOS.

The hs-CRP levels in the L-AGEs group decreased, although the change did not reach statistical significance. However, there was a significant reduction in TNF-a levels in the L-AGEs group. These results align with observations reported in prior studies [41–43]. Chao et al. [41], reported a direct relationship between elevated dietary intake of AGEs and TNF-α levels in individuals diagnosed with type 2 diabetes mellitus. Similarly, another study demonstrated that lowering dietary AGEs in healthy individuals resulted in decreased TNF-α levels [42]. The AGE-RAGE system activates intracellular pathways, causing an increase in reactive oxygen species (ROS), cellular oxidative stress, and an increase in TNF-α [43]. In this study, no significant change in oxidative stress index (OSI) was observed in both group. However, TAS levels decreased significantly in the S-AGEs group. Tantalaki et al.[19], reported that oxidative stress increased in individuals who followed a high AGEs diet for 2 months.

Insulin resistance has been linked to PCOS in a large proportion of this patient population, owing to oxidative stress and inflammation [44, 45]. Insulin resistance and hyperinsulinemia play well-established roles in endocrine and reproductive problems in PCOS. Excess androgens, especially in muscle and fat tissue, can impair insulin action [45]. In a study with 6 women and 14 men, matched for isoenergetic and macronutrient content, with high and low AGEs contents, were ingested every two weeks and interspersed with a four-week washout interval. An intravenous glucose tolerance test and a hyperinsulinemic-euglycemic clamp were conducted at the start and finish of each meal phase. Results showed that although a high dietary AGEs intake did not result in alterations in body weight and insulin secretion, it did cause a decline in insulin sensitivity [46]. Overweight women who followed a low AGEs diet for four weeks showed a notable decrease in fasting serum insulin levels and HOMA-IR compared to overweight women on a high AGEs diet [47]. A meta-analysis exploring the effects of low AGEs diets on cardiometabolic factors showed decreases in insulin resistance, total cholesterol, LDL, and TNF. Nevertheless, there were no changes noted in body weight, fasting glucose, or hemoglobin A1c levels [48]. In this study, the L-AGEs group showed significant decrease in fasting glucose levels, compared to the S-AGEs group. Fasting insulin levels and HOMA-IR reduced dramatically in both groups, most likely due to weight loss. Furthermore, QUCKI levels dropped considerably in the L-AGEs group. While weight loss appears to be an important aspect in resolving insulin resistance, the observed effect on fasting glucose levels suggests that reducing AGEs intake through dietary interventions, in addition to weight loss, may result in better outcomes.

The interaction with AGE-RAGE triggers signaling pathways, resulting in increased oxidative stress, inflammation, hyperandrogenism, ovulatory dysfunction, insulin resistance, and obesity. Furthermore, dietary AGEs may impair oocyte competence, healthy embryo development, and, eventually, impact pregnancy outcomes. Increased levels of serum AGEs in reproductive-age women could exacerbate reproductive dysfunction associated with PCOS. An expanding body of evidence suggests that the detrimental impact of the AGE-RAGE axis on folliculogenesis and the follicular microenvironment contributes to ovarian dysfunction and suboptimal IVF outcomes. Nevertheless, the underlying mechanisms of this association remain unclear [28, 38]. A 2019 systematic review, which included healthy obese individuals, indicated that weight loss through calorie restriction or bariatric surgery decreased serum AGEs and raised sRAGE [25].

Our study has several limitations. First, some participants in both study groups did not complete the clinical trial after random assignment. This is consistent with previous research on weight loss in PCOS, which has also reported low completion rates. The extended follow-up period likely contributed to the high dropout rate, a common issue in weight loss programs [49, 50]. Despite the dropout rate, there were no significant differences in age, anthropometric measurements, or metabolic indicators between those who completed the study and those who dropped out. Small sample size in groups, which may have prevented the detection of significant differences between groups. Second limitation of the present study, the analysis included only the women who completed the trial and the dietary data.

In conclusion, involving obese individuals diagnosed with PCOS while both diet groups in this study experienced similar weight loss, only the L-AGEs group demonstrated an improvement in serum AGE levels. Besides weight loss, lowering intake of dietary AGEs led to notable improvements in LDL cholesterol, TNF-A, total testosterone, SHBG, and AMH levels among PCOS phenotype A individuals. Furthermore, the progress noted in menstrual irregularity and m-FG scores holds promise for forthcoming outcomes. Reducing dietary AGEs, along with weight loss, may provide more significant improvements in hormonal parameters. Results seem promising in terms of recovering dysfunction.

## Data Availability

Data will be made available on request.
